# Exploring optimal methods for age-at-death estimation using pulp/tooth area ratios: a South African study

**DOI:** 10.1007/s00414-024-03360-7

**Published:** 2024-11-01

**Authors:** Daniël Kotze, Calvin G. Mole, Vincent M. Phillips, Victoria E. Gibbon

**Affiliations:** 1 Division of Clinical Anatomy and Biological Anthropology, Department of Human Biology, Faculty of Health Sciences, University of Cape Town, Private Bag X3, Observatory, Cape Town, 7935 South Africa; 2 Division of Forensic Medicine and Toxicology, Department of Pathology, University of Cape Town, Cape Town, South Africa; 3 Department of Oral Pathology and Forensic Sciences, Oral Health Centre, Faculty of Dentistry, University of the Western Cape, Cape Town, South Africa

**Keywords:** Forensic sciences, Legal medicine, Age estimation, Pulp/tooth area ratio, Dental radiography, Canine teeth

## Abstract

Age-at-death estimation is pivotal in the identification of unknown human decedents in forensic medicine. The pulp/tooth area ratio (PAR) method, assessing pulp cavity size as a marker of secondary dentine apposition, is widely utilised for adult age estimation. Despite extensive evaluation of this technique, the influence of image type and enamel area inclusion on method accuracy is insufficiently explored. The present study evaluated the PAR method’s applicability using maxillary canines from a South African cadaveric sample, examining reliability, sex bias, and accuracy across different image types and enamel area considerations. An observational, cross-sectional study design was followed. Fifty-two adult maxillary canines were radiographed, sectioned, and analysed using stereomicroscopy. Labiolingual and mesiodistal periapical radiographs, alongside labiolingual stereomicroscopic tooth section images, were analysed using ImageJ to calculate PARs. Age estimation linear regression models were developed for each image type, with and without enamel area inclusion. Models were compared for performance and accuracy using best-subsets regression and cross-validation analyses. Results indicated that the PAR method is a reliable, sex-independent technique, providing relatively accurate age-at-death estimates for South African adults. Image type significantly influenced accuracy, with tooth section images exhibiting the best/lowest error values, followed by labiolingual and mesiodistal radiographs, respectively. Exclusion of enamel area consistently enhanced model performance across all image types. This study contributes valuable data to the underexplored field of dental age estimation techniques for South African adults, highlighting optimal approaches when applying the PAR method to maxillary canines. Additionally, it introduces a refined stereomicroscopic technique, augmenting and enhancing existing practices.

**Supplementary Information:**

The online version contains supplementary material available at 10.1007/s00414-024-03360-7.

## Introduction

Skeletal age-at-death estimation is fundamental to biological anthropology, providing valuable insights into past population demographics, health, lifeways, and evolutionary trends [1, 2]. In forensic medicine and science, it plays a crucial role in the identification of unknown human decedents, contributing significantly to an individual’s biological profile, alongside sex, stature, and ancestry analyses [3, 4]. This information aids law enforcement in refining missing persons lists, thereby improving the efficiency of medico-legal investigations and identification efforts [5, 6]. Identifying deceased individuals holds profound ethical and judicial value, helping resolve various legal issues, deliver justice, and provide closure to affected families and communities [7–9].

Among various biological indicators, teeth emerge as reliable age markers due to their durability and resistance to taphonomic changes [10, 11]. Adult dental ageing methods rely on post-formation, regressive changes that typically exhibit greater variability and inaccuracy compared to subadult indicators [12]. These techniques encompass visual, histological, radiographic, biochemical and genetic/epigenetic approaches [13]. Radiographic methods, particularly those evaluating secondary dentine apposition, are valued for their efficiency, minimal invasiveness and relative accuracy [14]. Secondary dentine deposition occurs throughout life following root formation, gradually reducing a tooth’s pulp cavity, providing useful age-related information [15].

The *pulp/tooth area ratio* (PAR) method, pioneered by Cameriere et al. [16–18], is a widely utilised adult ageing technique, which quantifies changes in pulp cavity size from radiographs as an indirect measure of secondary dentine apposition. The PAR has shown strong correlation with chronological age, offering reliable, sex-independent and relatively accurate age estimates, with canine mean absolute errors as low as 2.43 years in certain populations [14, 19]. Despite nearly two decades of evaluation, several research gaps persist for the PAR method. Notably, studies on pulp/tooth volume ratios have shown better age estimation accuracy when excluding enamel dimensions from analyses [20, 21]; however, such research for PARs is lacking. Moreover, while labiolingual radiographic views are predominantly used for PAR assessments, the relative utility of mesiodistal views is inadequately investigated [18, 22]. Additionally, limited research has compared age estimates from radiographs versus tooth section images, often relying on published equations developed for radiographic data, which may introduce biases and compromise comparison validity [23, 24].

This study aimed to evaluate the applicability of the PAR method for estimating adult age-at-death using maxillary canines from a South African cadaveric sample. Objectives included (1) assessing the reliability of the PAR method, (2) examining sex differences in PAR values, (3) comparing the performance of the PAR method across different image types (labiolingual periapical radiographs, mesiodistal periapical radiographs, labiolingual stereomicroscopic tooth section images), and (4) contrasting age estimation models derived from PAR values with and without consideration of enamel areas. Such validation is critical to account for population-specific variations with ageing [25], contributing valuable data to the limited research on dental ageing techniques for South African adults [26, 27]. This study also provides insights for global practitioners on optimal approaches when utilising the PAR method for maxillary canines and introduces a modified stereomicroscopic technique.

## Materials & methods

Ethical clearance was granted by the Human Research Ethics Committee at the University of Cape Town (UCT) (HREC REF: 336/2022). Permission was obtained from UCT’s Cadaver Research Governance Committee (CRGC 2022/001), as well as the Division of Clinical Anatomy, Faculty of Medicine and Health Sciences, Stellenbosch University (SU), and the Provincial Inspector of Anatomy. This research, conducted during 2022 and 2023, complies with the STROBE protocol for observational, cross-sectional studies.

The sample included 52 individuals with known chronological age and sex (Table 1). The number of suitable teeth available during the study period determined the sample size. Maxillary canines were sourced from cadaveric human donor bodies at the Department of Human Biology, UCT (29 individuals) and the Division of Clinical Anatomy, SU (23 individuals). The sample comprised 25 males and 27 females, aged between 26 and 91 years (mean age ± standard deviation = 62.4 ± 19.9 years). According to age categories defined by Botha and Steyn [28], the sample included 32 older adults (≥ 60 years), 8 middle-aged adults (40–59 years) and 12 younger adults (16–39 years).

**Table 1:** Summary statistics for the sample demographic variables ─ sex and age (years)

Age category	Age interval	Males (*n*)	Females (*n*)	Pooled sexes (*n*)	Mean age ± SD
Younger adults	20–29	0	3	3	27.7 ± 1.5
	30–39	7	2	9	35.1 ± 3.5
Middle-aged adults	40–49	1	1	2	42.5 ± 3.5
	50–59	3	3	6	54.5 ± 2.6
Older adults	60–69	6	2	8	63.3 ± 2.9
	70–79	3	8	11	74.9 ± 2.9
	80–89	5	6	11	83.8 ± 3.1
	90–99	0	2	2	90.5 ± 0.7
Total		25	27	52	62.4 ± 19.9

n = number of individuals (sample size); SD = standard deviation

### Tooth sampling & selection

Aligned with Merdietio Boedi [29], only adults (≥ 18 years) with at least one fully erupted, mature (closed apex), and healthy maxillary canine were included. Previous research found no significant PAR measurement differences between the left and right sides [14]. Therefore, the right tooth was prioritised for analysis; the left was used when the right was unavailable or unsuitable. Teeth were excluded if they showed dental pathologies (e.g. caries and traumatic lesions), developmental anomalies (e.g. impaction and multiple roots), artificial modifications (e.g. restorative procedures) and/or severe dental wear, characterised by at least Phase G of the Lovejoy [30] scoring system.

### Radiography

Radiographic imaging was conducted using an Intra-oral X-ray Machine (ACTEON X-MIND), with exposure parameters: 70 kv; 8 mA; 0.50 ms. Periapical radiographs were obtained using indirect digital imaging, where the receptor was scanned (Intra-oral Scanner - Carestream CS7600) following exposure to X-radiation.

Each tooth was radiographed separately using the paralleling technique [31], capturing labiolingual and mesiodistal image projections. Positioned on a flat surface, each tooth was placed directly on a phosphor plate receptor and secured with Sellotape. This technique reduced the object-receptor distance, minimising magnification and loss of definition [31]. The cone (length = 167 mm) was centred over the flat surface, covering the phosphor plate and tooth, thereby standardising the target-receptor distance and limiting motion.

### Sectioning & stereomicroscopy

Radiographed teeth were sectioned for stereomicroscopic analysis. The ideal cutting plane was marked on the mesial and distal tooth surfaces prior to embedding each tooth individually in an epoxy resin block (Kristal50 [AMT Composites, Maitland, Cape Town]). Embedded teeth were sectioned longitudinally along the mesiodistal plane (non-obliquely through the crown’s long axis) using a Buehler Isomet saw with a diamond wafering blade (200 RPM, 3 mm/min). This produced thick labial and lingual portions, exposing the labiolingual view of the pulp cavity and surrounding tissue. This plane of sectioning was chosen as the mesiodistal view presented minimal root curvature and large pulp cavity dimensions, ensuring the consistent acquisition of complete tooth sections.

Tooth sections were examined under a Zeiss Discovery V20 stereomicroscope with an Axiocam 503 colour camera at 10x magnification. Each tooth was imaged in three parts and digitally stitched together using Hugin^®^ 2022.0.0 (open-source panorama photo stitcher, Pablo d’Angelo) to create a single composite image.

### Image analyses

**Fig. 1:**
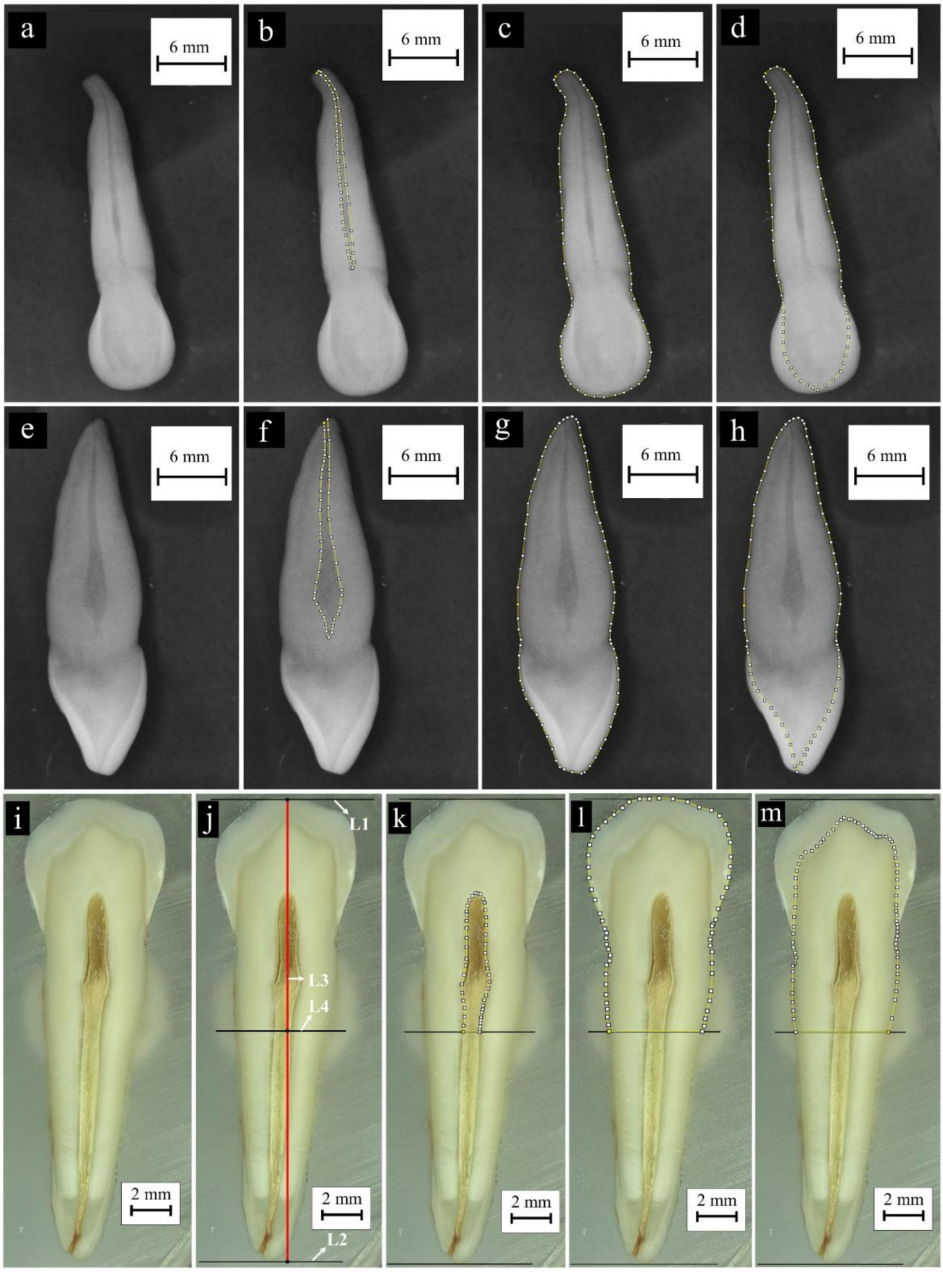
Labiolingual radiograph (top row), mesiodistal radiograph (middle row) and stereomicroscopic tooth section image (bottom row) measurements using ImageJ. **a**) unmeasured labiolingual radiograph, **b**) pulp cavity area measured from labiolingual radiograph, **c**) complete tooth area measured from labiolingual radiograph, **d**) tooth area (excluding enamel) measured from labiolingual radiograph, **e**) unmeasured mesiodistal radiograph, **f**) pulp cavity area measured from mesiodistal radiograph, **g**) complete tooth area measured from mesiodistal radiograph, **h**) tooth area (excluding enamel) measured from mesiodistal radiograph, **i**) unaltered stereomicroscopic tooth section image, **j**) lines (L1 – L4) drawn/positioned consecutively to systematically obtain the region of interest for conducting measurements, **k**) pulp cavity area measured from stereomicroscopic tooth section image, **l**) complete tooth area measured from stereomicroscopic tooth section image, m) tooth area (excluding enamel) measured from stereomicroscopic tooth section image

Radiographic and stereomicroscopic images were analysed using ImageJ 1.54b (open-source imaging processing program, National Institutes of Health, US). Following D’Ortenzio et al. [23], areas (mm²) were calculated using the polygon selection tool, with at least 50 points marked for each tooth outline (including and excluding the enamel) and 40 points for each pulp cavity outline (Fig. 1).

Due to the curvature and tapering of root canals towards the apex, observation of the entire pulp cavity in this region post-sectioning was not always feasible. Therefore, measurements were confined to the coronal half of each tooth section, systematically obtained as follows (Fig. 1): (1) Tooth sections were imaged with the crown’s long axis aligned approximately parallel to the vertical plane; (2) Two tangential horizontal lines (L1 and L2) were drawn along the most coronal and apical aspects of the tooth area outline; (3) A temporary vertical line (L3) connected L1 and L2; (4) A third horizontal line (L4) was drawn through the midpoint of L3, dividing the tooth into two portions; (5) Only the region coronal to L4 was considered for measurements.

### Statistical analyses

Unless noted otherwise, data analyses used the IBM^®^ SPSS^®^ Statistics software (version 28), with *p* ≤ 0.05 indicating statistical significance. PARs were calculated by dividing pulp cavity area by total tooth area (including and excluding enamel).

Intra- and inter-observer reliability were assessed by re-examination of 19 randomly selected images (of each type) two weeks after initial analysis. Inter-observer analyses were performed by a single individual with experience and training on the measurement technique. Agreement between raters was determined using the intraclass correlation coefficient (ICC) [32].

Analysis of covariance (ANCOVA) assessed sex effects on PARs, controlling for age. Pearson’s correlation was used to evaluate linearity between age and PARs based on criteria by Rowntree [33]. Moderation analysis via PROCESS macro (v4.2) in SPSS [34] explored interaction effects. Additionally, multicollinearity among predictors was assessed using the variance inflation factor (VIF > 5 indicates problematic multicollinearity) [35].

The relationship between age-at-death (response variable) and PARs (predictor variables) was evaluated through linear regression. Best-subsets regression, conducted in JMP^®^ 17.1 (SAS Institute Inc., Cary, NC, 1989–2023), compared age estimation models using statistical criteria, including the adjusted coefficient of determination (*R*^2^-adjusted), standard error of the estimate (SEE), Akaike information criterion adjusted for sample size (AIC_c_), Bayesian information criterion (BIC), and Mallows’ *C*_*p*_ statistic (*C*_*p*_) [36]. Models were selected based on superior performance across these criteria, with only the most effective ones advancing. Additional parameters, including the mean absolute error (MAE), coefficient of determination (*R*^2^), standard deviation (SD) of the absolute residuals, and a 95% prediction interval, were also reported. Additionally, models underwent a post hoc power analysis using G*Power 3.1.9.7 [37]. to ensure adequate power (≥ 0.80).

Selected models underwent a leave-one-out cross-validation (LOOCV), performed in RStudio^®^ 2023.03.1.446 using the *trainControl* function from the *caret* library [38]. Cross-validation parameters, including the coefficient of determination (*R*^2^_CV_), mean absolute error (MAE_CV_) and standard error of the estimate (SEE_CV_), were calculated to evaluate model performance. Optimism analysis, comparing cross-validation and in-sample results, gauged predictive bias.

A two-way repeated measures analysis of variance (ANOVA) (with two within-subjects factors) analysed effects of image type and enamel area on absolute estimate residuals/errors, followed by Fisher’s least significant difference procedure for multiple comparisons.

## Results

All variables demonstrated ICC estimates above 0.9 during both intra- and inter-observer reliability assessments, indicating excellent data reproducibility (Table 2). Age significantly correlated with all PAR variables in the ANCOVA (*p* < 0.001), while sex did not exhibit a significant effect on these variables (Table 3). Consequently, sex-specific age estimation models were not developed for the sample. The moderation analysis did not reveal any significant first-order interaction effects among predictor variables (see Online Resource 1).

**Table 2:** Observer reliability assessment results for each variable

Inter-observer reliability				Intra-observer reliability			
Variables	ICC	95% CI		Variables	ICC	95% CI	
		Lower bound	Upper bound			Lower bound	Upper bound
PA_1_	0.94	0.66	0.98	PA_1_	0.99	0.98	1.00
TA_1_	1.00	1.00	1.00	TA_1_	1.00	1.00	1.00
TA_EE_1_	0.99	0.97	1.00	TA_EE_1_	1.00	0.99	1.00
PAR_1_	0.92	0.50	0.98	PAR_1_	0.99	0.98	1.00
PAR_EE_1_	0.93	0.67	0.98	PAR_EE_1_	0.99	0.98	1.00
PA_2_	0.95	0.62	0.99	PA_2_	0.98	0.95	0.99
TA_2_	1.00	1.00	1.00	TA_2_	0.95	0.88	0.98
TA_EE_2_	1.00	0.98	1.00	TA_EE_2_	0.94	0.85	0.98
PAR_2_	0.92	0.47	0.98	PAR_2_	0.96	0.89	0.98
PAR_EE_2_	0.94	0.60	0.98	PAR_EE_2_	0.95	0.88	0.98
PA_3_	0.99	0.94	1.00	PA_3_	1.00	1.00	1.00
TA_3_	0.98	0.95	0.99	TA_3_	1.00	1.00	1.00
TA_EE_3_	0.97	0.91	0.99	TA_EE_3_	1.00	1.00	1.00
PAR_3_	0.99	0.91	1.00	PAR_3_	1.00	1.00	1.00
PAR_EE_3_	0.99	0.97	1.00	PAR_EE_3_	1.00	1.00	1.00

ICC = intraclass correlation coefficient; CI = confidence interval; PA_1_ = pulp cavity area measured from labiolingual radiograph; TA_1_ = complete tooth area measured from labiolingual radiograph; TA_EE_1_ = tooth area (excluding enamel) measured from labiolingual radiograph; PAR_1_ = pulp/tooth area ratio obtained from labiolingual radiograph; PAR_EE_1_ = pulp/tooth area ratio (excluding enamel) obtained from labiolingual radiograph; PA_2_ = pulp cavity area measured from mesiodistal radiograph; TA_2_ = complete tooth area measured from mesiodistal radiograph; TA_EE_2_ = tooth area (excluding enamel) measured from mesiodistal radiograph; PAR_2_ = pulp/tooth area ratio obtained from mesiodistal radiograph; PAR_EE_2_ = pulp/tooth area ratio (excluding enamel) obtained from mesiodistal radiograph; PA_3_ = pulp cavity area measured from stereomicroscopic tooth section image; TA_3_ = complete tooth area measured from stereomicroscopic tooth section image; TA_EE_3_ = tooth area (excluding enamel) measured from stereomicroscopic tooth section image; PAR_3_ = pulp/tooth area ratio obtained from stereomicroscopic tooth section image; PAR_EE_3_ = pulp/tooth area ratio (excluding enamel) obtained from stereomicroscopic tooth section image

**Table 3:** Analysis of covariance (ANCOVA) results for assessing the main effect of sex on the pulp/tooth area ratio (PAR) variables, with age as the covariate

Variables	df	SS	MS	F-statistic	*p*
**PAR**_**1**_					
Age	1	0.02	0.02	83.63	< 0.001
Sex	1	3.15e^− 4^	3.15e^− 4^	1.34	0.253
Residuals	49	0.01	2.36e^− 4^		
**PAR_EE**_**1**_					
Age	1	0.03	0.03	86.88	< 0.001
Sex	1	9.67e^− 5^	9.67e^− 5^	0.31	0.579
Residuals	49	0.02	3.10e^− 4^		
**PAR**_**2**_					
Age	1	0.03	0.03	32.79	< 0.001
Sex	1	3.68e^− 3^	3.68e^− 3^	3.74	0.059
Residuals	49	0.05	9.86e^− 4^		
**PAR_EE**_**2**_					
Age	1	0.04	0.04	35.03	< 0.001
Sex	1	3.31e^− 3^	3.31e^− 3^	2.72	0.105
Residuals	49	0.06	1.22e^− 3^		
**PAR**_**3**_					
Age	1	0.04	0.04	124.13	< 0.001
Sex	1	2.16e^− 4^	2.16e^− 4^	0.76	0.386
Residuals	49	0.01	2.83e^− 4^		
**PAR_EE**_**3**_					
Age	1	0.06	0.06	148.35	< 0.001
Sex	1	1.86e^− 5^	1.86e^− 5^	0.05	0.828
Residuals	49	0.02	3.92e^− 4^		

PAR_1_ = pulp/tooth area ratio obtained from labiolingual radiograph; PAR_EE_1_ = pulp/tooth area ratio (excluding enamel) obtained from labiolingual radiograph; PAR_2_ = pulp/tooth area ratio obtained from mesiodistal radiograph; PAR_EE_2_ = pulp/tooth area ratio (excluding enamel) obtained from mesiodistal radiograph; PAR_3_ = pulp/tooth area ratio obtained from stereomicroscopic tooth section image; PAR_EE_3_ = pulp/tooth area ratio (excluding enamel) obtained from stereomicroscopic tooth section image; df = degrees of freedom; SS = sum of squares; MS = mean square

**Fig. 2:**
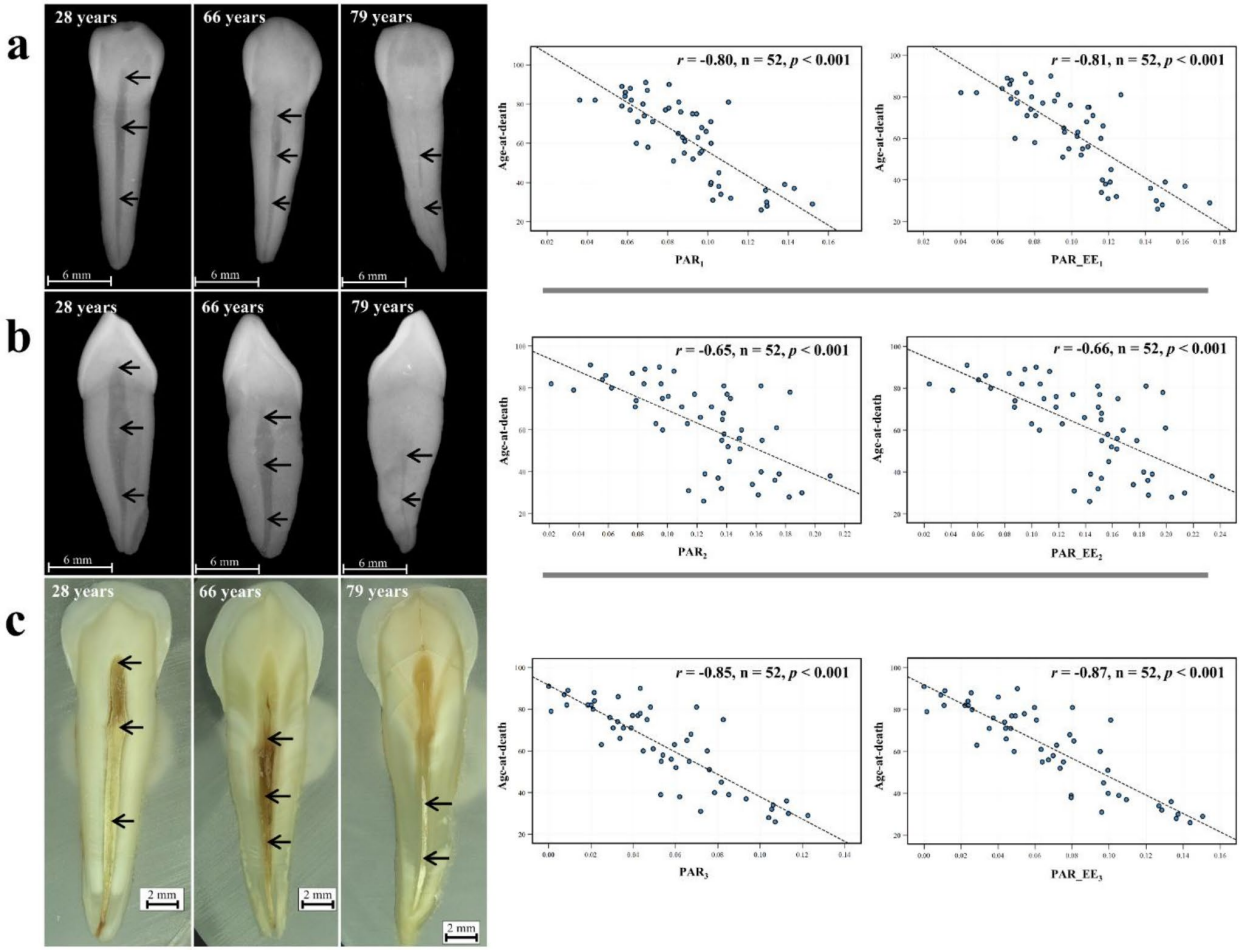
Age-related changes in maxillary canine pulp/tooth area ratios (PARs) observed from labiolingual periapical radiographs (**a**), mesiodistal periapical radiographs (**b**) and labiolingual stereomicroscopic tooth section images (**c**). For the dental images on the left side, each column shows three different image types from one individual, while each row shows three of the same image types from individuals of different ages. On the right side, scatter plots show the direction and strength of the linear relationship between age-at-death and the PAR variables. The Pearson correlation coefficient (*r*) and associated statistics are reported on each graph. As individuals age, their pulp cavities (indicated by the arrows) become increasingly obliterated, reducing the ratio of pulp cavity to tooth area. PAR_1_ = pulp/tooth area ratio obtained from labiolingual radiograph; PAR_EE_1_ = pulp/tooth area ratio (excluding enamel) obtained from labiolingual radiograph; PAR_2_ = pulp/tooth area ratio obtained from mesiodistal radiograph; PAR_EE_2_ = pulp/tooth area ratio (excluding enamel) obtained from mesiodistal radiograph; PAR_3_ = pulp/tooth area ratio obtained from stereomicroscopic tooth section image; PAR_EE_3_ = pulp/tooth area ratio (excluding enamel) obtained from stereomicroscopic tooth section image; n = sample size

Pearson correlation coefficients and scatter plots indicated a moderate to strong negative linear relationship between PAR variables and age (Fig. 2). Stereomicroscopic tooth section images showed the strongest correlation values (*r* = -0.85 and − 0.87), followed by labiolingual radiographs (*r* = -0.80 and − 0.81) and mesiodistal radiographs (*r* = -0.65 and − 0.66), respectively. Notably, for each image type, PAR variables excluding enamel area exhibited stronger correlations with age-at-death. Figure 2 illustrates age-related changes in maxillary canine PARs: as individuals age, their pulp cavities become increasingly obliterated, thereby reducing the ratio of pulp cavity to tooth area. Summary statistics for the PAR variables are provided in Online Resource 1.

### Model selection

Employing a best-subsets regression approach, 63 candidate models were initially developed from the six PAR predictor variables. Models with high multicollinearity (VIF values > 5, see Online Resource 1) were first eliminated. Based on the selection criteria, 14 suitable models were chosen for age estimation (Table 4). These included six simple and eight multiple (incorporating two predictor variables) linear regression models (see Online Resource 1 for ANOVA and coefficients table outputs).

Among the simple regression models, those derived from stereomicroscopic tooth section images showed superior performance for age estimation, followed by models from labiolingual and mesiodistal radiographs, respectively (Table 4). Notably, models excluding enamel area consistently outperformed those including enamel within each image type group. Some multiple regression models combining PARs from labiolingual radiographs and stereomicroscopic tooth section images, as well as labiolingual and mesiodistal radiographs, demonstrated enhanced performance compared to simpler models.

Models based on stereomicroscopic tooth section images achieved the best MAE (7.21–7.47 years), SEE (9.88–10.53 years) and *R*^2^ (0.73–0.76) values. Models derived from labiolingual radiographs followed (MAE = 9.52–9.76 years; SEE = 11.90–12.03 years; *R*^2^ = 0.64–0.65), with those derived from mesiodistal radiographs performing less accurately (MAE = 11.87–11.93 years; SEE = 15.11–15.33 years; *R*^2^ = 0.42–0.44) (Tables 4 and 5). Within each image type group, the model excluding enamel area showed superior MAE, SEE and *R*^2^ values.

Most selected multiple regression models exhibited minor accuracy improvements (less than one year) over corresponding simpler models (Tables 4 and 5). Models combining data from stereomicroscopic tooth section images and labiolingual radiographs achieved MAEs and SEEs of 6.97–7.47 years and 9.65–10.20 years, respectively, with *R*^2^ ranging between 0.75 and 0.77. Those combining PARs from labiolingual and mesiodistal radiographs demonstrated MAEs and SEEs of 9.13–9.20 years and 11.76–11.92 years, respectively, while *R*^2^ ranged between 0.66 and 0.67.

The 95% prediction interval ranged from ± 19.85 years to ± 30.79 years for simple regression models and ± 19.40 years to ± 23.95 years for multiple regression models (Table 5). Residuals from all models were normally distributed with equal variance according to Shapiro-Wilk and Breusch-Pagan tests (see Online Resource 1). Additionally, all models demonstrated large effect sizes and sufficient statistical power (≥ 0.80) (Table 5).

**Table 4:** Best-subsets regression results for all suitable models chosen. Models are grouped and ranked separately based on the number of predictor variables (k) they contain. Within each size group (k), they are ordered based on performance – from top (best) to bottom (worst) – as determined by selection criteria values

k	Image type	Model	Performance/selection criteria				
			*R*^2^-adjusted	SEE	AIC_c_	BIC	*C*_*p*_
1	**STSI**	Age = 91.83 – 439.05(PAR_EE_3_)	0.75	9.88	390.25	395.60	5.49
		Age = 91.47 – 534.64(PAR_3_)	0.72	10.53	396.86	402.22	12.74
	**LR**	Age = 117.77 – 548.95(PAR_EE_1_)	0.64	11.90	409.55	414.91	29.53
		Age = 118.29 – 625.44(PAR_1_)	0.64	12.03	410.74	416.10	31.32
	**MR**	Age = 100.82 – 281.03(PAR_EE_2_)	0.42	15.11	434.46	439.81	77.16
		Age = 100.24 – 307.84(PAR_2_)	0.41	15.33	435.91	441.27	80.72
2	**LR** **+** **STSI**	Age = 101.32 – 161.82(PAR_EE_1_) – 337.20(PAR_EE_3_)	0.77	9.65	389.11	396.06	4.01
		Age = 100.84 – 171.53(PAR_1_) – 344.89(PAR_EE_3_)	0.76	9.70	389.63	396.58	4.52
		Age = 103.68 – 208.15(PAR_EE_1_) – 373.11(PAR_3_)	0.74	10.09	393.77	400.73	8.71
		Age = 102.88 – 218.35(PAR_1_) – 385.67(PAR_3_)	0.74	10.20	394.88	401.84	9.89
	**LR** **+** **MR**	Age = 119.39 – 464.89(PAR_EE_1_) – 73.82(PAR_EE_2_)	0.65	11.76	409.65	416.61	28.25
		Age = 119.48 – 470.92(PAR_EE_1_) – 77.95(PAR_2_)	0.65	11.78	409.82	416.77	28.48
		Age = 119.84 – 524.76(PAR_1_) – 77.17(PAR_EE_2_)	0.64	11.88	410.68	417.64	29.73
		Age = 119.84 – 534.30(PAR_1_) – 78.91(PAR_2_)	0.64	11.92	411.03	417.98	30.24

STSI = stereomicroscopic tooth section image; LR = labiolingual radiograph; MR = mesiodistal radiograph; PAR_1_ = pulp/tooth area ratio obtained from labiolingual radiograph; PAR_EE_1_ = pulp/tooth area ratio (excluding enamel) obtained from labiolingual radiograph; PAR_2_ = pulp/tooth area ratio obtained from mesiodistal radiograph; PAR_EE_2_ = pulp/tooth area ratio (excluding enamel) obtained from mesiodistal radiograph; PAR_3_ = pulp/tooth area ratio obtained from stereomicroscopic tooth section image; PAR_EE_3_ = pulp/tooth area ratio (excluding enamel) obtained from stereomicroscopic tooth section image; *R*^2^-adjusted = adjusted coefficient of determination; SEE = standard error of the estimate; AIC_c_ = Akaike information criterion adjusted based on sample size; BIC = Bayesian information criterion; *C*_*p*_ = Mallows’ prediction criterion

**Table 5:** Accuracy and other performance parameters associated with each age estimation model selected during the best-subsets regression analysis. Models are listed in the same order as they appear in table 4

k	Image type	Model	*R* ^2^	*r*	*f* ^2^	Power	Absolute residual parameters (years)			95% PI (years)
							MAE	SD	MAE 95% CI	
1	**STSI**	Age = f(PAR_EE_3_)	0.76	-0.87	3.17	1.00	7.21	6.60	(5.37, 9.05)	± 19.85
		Age = f(PAR_3_)	0.73	-0.85	2.70	1.00	7.47	7.27	(5.45, 9.50)	± 21.15
	**LR**	Age = f(PAR_EE_1_)	0.65	-0.81	1.86	1.00	9.52	6.87	(7.61, 11.44)	± 23.89
		Age = f(PAR_1_)	0.64	-0.80	1.78	1.00	9.76	6.76	(7.88, 11.64)	± 24.17
	**MR**	Age = f(PAR_EE_2_)	0.44	-0.66	0.79	1.00	11.87	9.05	(9.35, 14.39)	± 30.36
		Age = f(PAR_2_)	0.42	-0.65	0.72	1.00	11.93	9.33	(9.33, 14.53)	± 30.79
2	**STSI** **+** **LR**	Age = f(PAR_EE_1_, PAR_EE_3_)	0.77	-0.88	3.35	1.00	6.97	6.45	(5.18, 8.77)	± 19.40
		Age = f(PAR_1_, PAR_EE_3_)	0.77	-0.88	3.35	1.00	7.03	6.45	(5.23, 8.83)	± 19.49
		Age = f(PAR_EE_1_, PAR_3_)	0.75	-0.87	3.00	1.00	7.40	6.62	(5.56, 9.24)	± 20.29
		Age = f(PAR_1_, PAR_3_)	0.75	-0.86	3.00	1.00	7.47	6.70	(5.60, 9.33)	± 20.50
	**LR** **+** **MR**	Age = f(PAR_EE_1_, PAR_EE_2_)	0.67	-0.82	2.03	1.00	9.13	7.06	(7.17, 11.10)	± 23.63
		Age = f(PAR_EE_1_, PAR_2_)	0.66	-0.81	1.94	1.00	9.14	7.07	(7.17, 11.11)	± 23.67
		Age = f(PAR_1_, PAR_EE_2_)	0.66	-0.81	1.94	1.00	9.16	7.21	(7.15, 11.17)	± 23.87
		Age = f(PAR_1_, PAR_2_)	0.66	-0.81	1.94	1.00	9.20	7.22	(7.19, 11.21)	± 23.95

k = number of predictor variables in the model; STSI = stereomicroscopic tooth section image; LR = labiolingual radiograph; MR = mesiodistal radiograph; *R*^2^ = coefficient of determination; *r* = correlation coefficient; *f*^2^ = Cohen’s effect size; MAE = mean absolute error; SD = standard deviation; CI = confidence interval; PI = prediction interval; PAR_1_ = pulp/tooth area ratio obtained from labiolingual radiograph; PAR_EE_1_ = pulp/tooth area ratio (excluding enamel) obtained from labiolingual radiograph; PAR_2_ = pulp/tooth area ratio obtained from mesiodistal radiograph; PAR_EE_2_ = pulp/tooth area ratio (excluding enamel) obtained from mesiodistal radiograph; PAR_3_ = pulp/tooth area ratio obtained from stereomicroscopic tooth section image; PAR_EE_3_ = pulp/tooth area ratio (excluding enamel) obtained from stereomicroscopic tooth section image

### Cross-validation

**Fig. 3:**
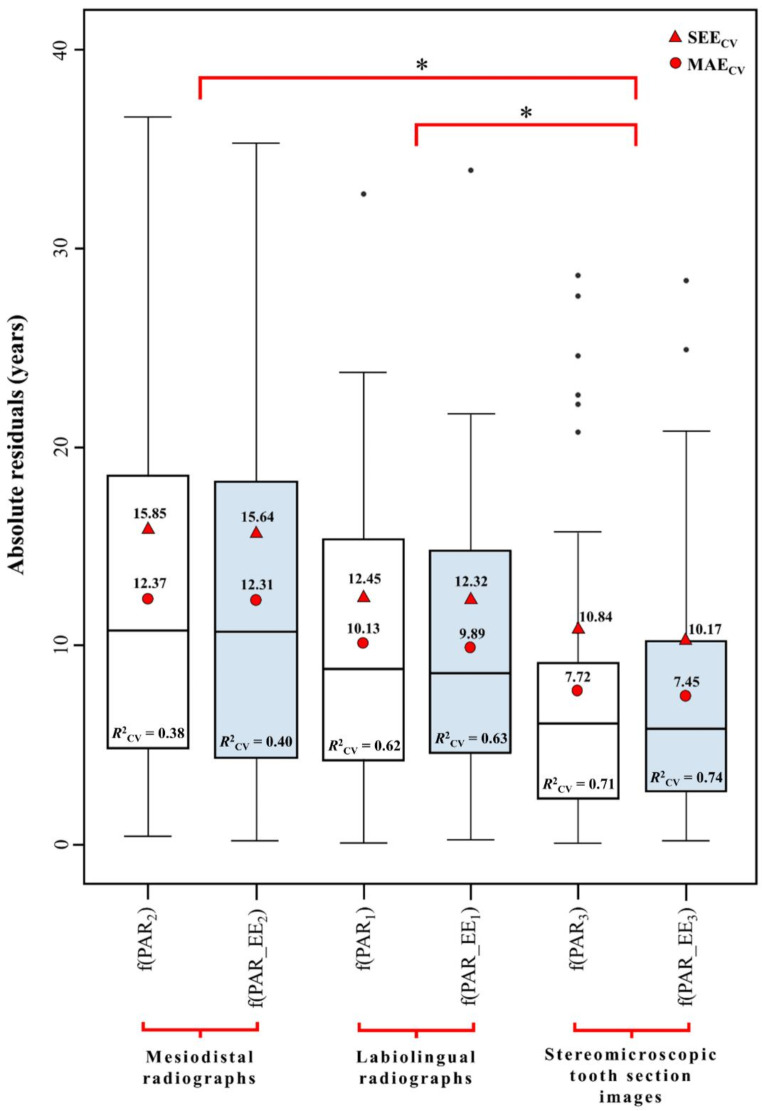
Box plots comparing the cross-validation absolute residual distributions associated with the simple linear regression age estimation models. Shaded box plots represent models excluding enamel. The cross-validation mean absolute error (MAE_CV_) and standard error of the estimate (SEE_CV_) (measured in years) associated with each model are shown as red circles and triangles for comparison. Each model’s cross-validation coefficient of determination (*R*^2^_CV_) is also indicated on the plots. Brackets with * indicate a significant difference (*p* ≤ 0.05) in MAE_CV_ between image type groups as determined by pairwise comparisons. PAR_1_ = pulp/tooth area ratio obtained from labiolingual radiograph; PAR_EE_1_ = pulp/tooth area ratio (excluding enamel) obtained from labiolingual radiograph; PAR_2_ = pulp/tooth area ratio obtained from mesiodistal radiograph; PAR_EE_2_ = pulp/tooth area ratio (excluding enamel) obtained from mesiodistal radiograph; PAR_3_ = pulp/tooth area ratio obtained from stereomicroscopic tooth section image; PAR_EE_3_ = pulp/tooth area ratio (excluding enamel) obtained from stereomicroscopic tooth section image

The cross-validation results are presented in Fig. 3, with detailed results provided in Online Resource 1. Minor differences in MAE and SEE values (below one year) were observed between in-sample and cross-validation assessments, indicating a relatively small prediction bias across all models. As before, models derived from stereomicroscopic tooth section images showed the best MAE_CV_ (7.45–7.72 years), SEE_CV_ (10.17–10.84 years) and *R*^2^_CV_ (0.71–0.74) values. Models derived from labiolingual radiographs followed (MAE_CV_ = 9.89–10.13 years; SEE_CV_ = 12.32–12.45 years; *R*^2^_CV_ = 0.62–0.63), while those derived from mesiodistal radiographs performed least effectively (MAE_CV_ = 12.31–12.37 years; SEE_CV_ = 15.64–15.85 years; *R*^2^_CV_ = 0.38–0.40). Exclusion of enamel area consistently improved model performance across all image types.

Only two of the selected multiple regression models, derived from stereomicroscopic tooth section images and labiolingual radiographs, demonstrated similar or improved MAE_CV_, SEE_CV_ and *R*^2^_CV_ parameters compared to their simpler subset models (see Online Resource 1). However, improvements were marginal.

A complementary two-way repeated measures ANOVA evaluated the main and interaction effects of image type and enamel area on absolute estimate residuals (Table 6). Sphericity was observed for both image type and the interaction term (Mauchly’s test, *p* > 0.05). There was no significant interaction effect between image type and enamel area on absolute residuals (*F* = 0.44, df = 2, *p* = 0.643). A significant main effect was observed for image type (*F* = 7.48, df = 2, *p* < 0.001) but not for enamel area (*F* = 1.50, df = 1, *p* = 0.227). Pairwise comparisons (Table 7) showed that models derived from stereomicroscopic tooth section images had significantly lower mean absolute residuals compared to those derived from labiolingual (*p* = 0.026, 95% CI = [0.31, 4.55]) and mesiodistal radiographs (*p* = 0.001, 95% CI = [1.94, 7.57]) (Fig. 3). Models derived from labiolingual radiographs exhibited notably lower mean absolute residuals than those from mesiodistal radiographs, although this difference did not reach significance (*p* = 0.060, 95% CI = [-0.10, 4.76]).

**Table 6:** Tests of within-subjects effects results (sphericity assumed) for the two-way repeated measures analysis of variance (with two within-subjects factors)

Source	df	SS	MS	F-statistic	*p*
Image type	2	1176.13	588.06	7.48	< 0.001*
Error (image type)	102	8024.14	87.01	-	-
Enamel area	1	2.86	2.86	1.50	0.227
Error (enamel area)	51	97.16	1.91	-	-
Interaction	2	0.67	0.34	0.44	0.643
Error (interaction)	102	77.18	0.79	-	-

df = degrees of freedom; SS = sum of squares; MS = mean square. Values that meet statistical significance (*p* ≤ 0.05) are denoted by *

**Table 7:** Pairwise comparisons (using Fisher’s least significant difference procedure) results following the two-way repeated measures analysis of variance (with two within-subjects factors)

(I) Image type	(J) Image type	Mean difference (I – J)	SE	*p*	95% CI for difference
LR	MR	-2.33	1.21	0.060	(-4.76, 0.10)
	STSI	2.43	1.06	0.026*	(0.31, 4.55)
MR	LR	2.33	1.21	0.060	(-0.10, 4.76)
	STSI	4.76	1.40	0.001*	(1.94, 7.57)
STSI	LR	-2.43	1.06	0.026*	(-4.55, -0.31)
	MR	-4.76	1.40	0.001*	(-7.57, -1.94)

LR = labiolingual radiograph; MR = mesiodistal radiograph; STSI = stereomicroscopic tooth section image; SE = standard error; CI = confidence interval. Values that meet statistical significance (*p* ≤ 0.05) are denoted by *

## Discussion

This study evaluated the PAR method’s applicability for predicting adult age-at-death using maxillary canines from a South African cadaveric sample. We assessed the method’s reliability, sex bias, and accuracy across different image types, with and without consideration of enamel.

The PAR method demonstrated excellent intra- and inter-observer reliability. Its simplicity and reliance on direct observation quantification and computer-assisted image analysis reduces observer variability, distinguishing it from conventional, more subjective skeletal ageing techniques [6].

Our findings indicated non-significant sex differences in PARs, suggesting the potential for developing unified age estimation models across sexes. This aligns with prior research [14], supporting the method’s applicability for cases where skeletal sex estimation is challenging/impossible [10].

PAR models derived from labiolingual stereomicroscopic tooth section images were most effective for age estimation and produced significantly lower absolute estimate residuals/errors than models derived from radiographs. Aligned with D’Ortenzio et al. [23], this highlights the superior image resolution/sharpness and direct observation capabilities of stereomicroscopic images. Conversely, radiograph resolution and contrast were limited by indirect observations (via X-radiation) and other potential factors such as the machine focal spot size and pixel/crystal size of the receptor [39]. Additionally, two-dimensional radiographs are susceptible to dimensional distortion [31]. The non-linear morphology of maxillary canines introduces an angular relationship between their surfaces and the receptor, likely exacerbating image distortion [31].

Couoh and Bautista [24] and Keerthi Priyadharshini [40] observed non-significant differences between age estimates from radiographs and tooth section images. They suggested that incomplete dental sections resulting from root curvature (asymmetry) may have affected the accuracy of their results. We addressed this limitation by developing a modified stereomicroscopic technique focused on mesiodistal sectioning and measurements confined to the coronal half of each tooth section (discussed in the methods section). This adjusted approach allowed for superior age estimation accuracy from maxillary canine tooth section images. One limitation is that older adults or those with premature dentine apposition may exhibit pulp cavities that have receded (apically) beyond the coronal half of the tooth. However, this was only observed for the oldest individual in our sample (91 years), who was assigned a PAR value of zero. This suggests that the method can still discriminate age-at-death well into old age, likely due to relatively slow rates of secondary dentine deposition in canines [41, 42].

PAR models derived from labiolingual radiographs performed better than those based on mesiodistal images. Two factors may explain these results. Firstly, maxillary canine roots typically angle distally relative to the vertical crown axis [43], likely causing greater angular distortion during the paralleling procedure for mesiodistal radiographs [31]. Secondly, reductions in pulp cavity size primarily occur in a mesial-distal direction [44], visible in labiolingual views. In contrast, reductions in labial-lingual/vestibular-oral directions (observed in mesiodistal views) occur later in life (around 60–70 years) due to fibrous dentine formation [44], thereby weakening the correlation between age-at-death and PARs from mesiodistal radiographs.

While enamel area did not significantly affect estimate residuals, its exclusion consistently improved model performance across all image types, supporting similar studies on pulp/tooth volume ratios [20, 21]. Dental wear is a multifaceted and variable process [45, 46], likely introducing errors for age analyses dependent on tooth size. Our findings may suggest that excluding enamel area minimises this variability, enhancing accuracy in age estimation models. However, further research is required to assess the complex effect of tooth wear on PAR age estimates [24].

Overall, this research suggests that the PAR method is a reliable, sex-independent technique, providing relatively accurate age-at-death estimates for South African adults. It demonstrates superior accuracy compared to other dental ageing methods validated in this population, such as those developed by Gustafson [47] and Lamendin et al. [48], which have shown complete-sample MAEs ranging from 11.6 to 15.10 years [26, 27]. Age estimation of decedents in South Africa is problematic as most available methods were created using populations from the global north and are known to be relatively inaccurate locally [49–51]. South Africa is considered an epicentre for understanding human origins and has an exceptionally high rate of unidentified medico-legal decedents [9, 52–54]; therefore, local forensic and bioarchaeological applications for this method are extensive.

Our findings underscore the importance of image type and enamel area considerations for optimising method accuracy. We recommend excluding enamel area from PAR calculations to minimise errors from dental wear and enhance formula applicability across diverse population groups. Both labiolingual stereomicroscopic tooth section images and labiolingual radiographs proved effective for PAR-based age estimation, producing error values (MAE and/or SEE) of approximately 10 years or less – a recommended benchmark for age estimation methods [11, 15].

While relatively accurate, the PAR method produced broad 95% prediction intervals (± 20 years at best), a common limitation in adult age estimation techniques [6]. This imprecision reflects the complex relationship between biological and chronological age, shaped by individual and population differences in genetics, environment, health and lifestyle [52, 55]. As individuals age, variability in degenerative skeletal indicators increases, making precise predictions challenging for older adults and limiting the utility of biological profiles in forensic investigations. To improve precision, the PAR method should be used alongside other skeletal techniques and holistic analyses that incorporate a wider range of age-informative traits. Future research should also explore whether this method is more precise in younger adults and if separating analyses into life phases could enhance its utility.

While the stereomicroscopic approach delivered superior results, its invasive and time-consuming nature may limit practical application. Destructive sampling of human remains in forensic and archaeological sciences poses ethical and practical challenges, particularly regarding skeletal preservation [56–59]. This can impact future research opportunities, curation, the integrity of medicolegal evidence and the cultural/religious concerns of affected communities [56–59]. Practitioners should weigh the value of information obtainable from such techniques against feasibility on a case-by-case basis, considering factors like finances, resource availability, timeframe, procedural viability, and the ability to record and preserve other skeletal information prior to sampling [57, 59]. It is also important to explore alternative, minimally destructive methods that can provide comparable data.

Validating our sample-specific formulae across other South African cohorts is required to assess their generalisability. Multiple regression models were explored but marginally improved estimates and involved multiple procedures; hence, their application is not recommended. The sample age distribution was skewed towards older adults, which may diminish the predictive power of regression models for younger age groups. Future studies should increase the reference sample size to enhance variation and ensure balanced representation across age groups, thereby reducing prediction biases and optimising regression model performance [60]. Evaluation of the method, including the modified stereomicroscopic approach, across other tooth types and population groups is encouraged. The development of sample-specific formulae for non-South African population groups is recommended to account for population differences in skeletal ageing [25].

## Electronic supplementary material

Below is the link to the electronic supplementary material.

Supplementary Material 1

## Data Availability

Data are available upon reasonable request to the authors.
