# Supplementary material for: Exploring optimal methods for age-at-death estimation using pulp/tooth area ratios: a South African study
Source: Int J Legal Med. 2024 Nov 1;139(2):887–99. doi: 10.1007/s00414-024-03360-7 (PMC11850563; doi:10.1007/s00414-024-03360-7)
Supplement: Supplementary file 1 — Supplementary Material 1 [file 414_2024_3360_MOESM1_ESM.docx]

**Exploring optimal methods for age-at-death estimation using pulp/tooth area ratios: A South African study**

*International Journal of Legal Medicine*

D. Kotze^1^, C.G. Mole^2^, V.M. Phillips^3^, V.E. Gibbon^1^

^1^ Division of Clinical Anatomy and Biological Anthropology, Department of Human Biology, University of Cape Town, Cape Town, South Africa.

^2^ Division of Forensic Medicine and Toxicology, Department of Pathology, University of Cape Town, Cape Town, South Africa.

^3^ Department of Oral Pathology and Forensic Sciences, Oral Health Centre, Faculty of Dentistry, University of the Western Cape. (Retired) Professor Emeritus.

**Corresponding author: Victoria E. Gibbon**

Postal address: Department of Human Biology, Faculty of Health Sciences, University of Cape Town, Private Bag X3, Observatory, 7935, Cape Town, South Africa.

Phone: +27 21 4066235

Fax: +27 21 4487226

Email: victoria.gibbon@uct.ac.za

**Table S1** Results of the moderation analysis using hierarchical regression to assess first-order interaction effects. The variance inflation factor (VIF) is also shown for each pair combination of predictor variables, where values > 5 are indicative of high/problematic multicollinearity

| Interaction variable | ∆*R*^2^ | *F*-statistic | df1 | df2 | *p* | VIF |
| --- | --- | --- | --- | --- | --- | --- |
| PAR_3_ * PAR_EE_3_ | < 0.01 | 0.59 | 1 | 48 | 0.446 | 90.44 |
| PAR_EE_1_ * PAR_EE_3_ | < 0.01 | 0.46 | 1 | 48 | 0.499 | 3.61 |
| PAR_1_ * PAR_EE_3_ | < 0.01 | 0.49 | 1 | 48 | 0.489 | 3.60 |
| PAR_EE_2_ * PAR_EE_3_ | 0.01 | 3.05 | 1 | 48 | 0.087 | 2.06 |
| PAR_2_ * PAR_EE_3_ | 0.01 | 2.72 | 1 | 48 | 0.105 | 2.01 |
| PAR_EE_1_ * PAR_3_ | < 0.01 | 0.30 | 1 | 48 | 0.588 | 3.43 |
| PAR_1_ * PAR_3_ | < 0.01 | 0.32 | 1 | 48 | 0.576 | 3.57 |
| PAR_EE_2_ * PAR_3_ | 0.01 | 2.67 | 1 | 48 | 0.109 | 2.04 |
| PAR_2_ * PAR_3_ | 0.01 | 2.26 | 1 | 48 | 0.139 | 2.02 |
| PAR_EE_1_ * PAR_EE_2_ | 0.01 | 1.52 | 1 | 48 | 0.223 | 2.03 |
| PAR_EE_1_ * PAR_2_ | 0.01 | 1.56 | 1 | 48 | 0.218 | 1.96 |
| PAR_1_ * PAR_EE_2_ | 0.01 | 1.55 | 1 | 48 | 0.220 | 2.03 |
| PAR_1_ * PAR_2_ | 0.01 | 1.44 | 1 | 48 | 0.235 | 1.98 |
| PAR_1_ * PAR_EE_1_ | < 0.01 | 0.35 | 1 | 48 | 0.559 | 117.35 |
| PAR_2_ * PAR_EE_2_ | < 0.01 | 0.16 | 1 | 48 | 0.693 | 204.37 |
| PAR_1_ = pulp/tooth area ratio obtained from labiolingual radiograph; PAR_EE_1_ = pulp/tooth area ratio (excluding enamel) obtained from labiolingual radiograph; PAR_2_ = pulp/tooth area ratio obtained from mesiodistal radiograph; PAR_EE_2_ = pulp/tooth area ratio (excluding enamel) obtained from mesiodistal radiograph; PAR_3_ = pulp/tooth area ratio obtained from stereomicroscopic tooth section image; PAR_EE_3_ = pulp/tooth area ratio (excluding enamel) obtained from stereomicroscopic tooth section image; ∆*R*^2^ = change in the coefficient of determination explained by the interaction effect; df = degrees of freedom. The * indicates an interaction between two predictor variables. | | | | | | |

**Table S2** Summary statistics for the pulp/tooth area ratio (PAR) predictor variables derived from each image type from the study sample

| Labiolingual radiographs | | Mesiodistal radiographs | | Stereomicroscopic  tooth section images | |
| --- | --- | --- | --- | --- | --- |
| Mean ± SD PAR_1_ | Mean ± SD PAR_EE_1_ | Mean ± SD PAR_2_ | Mean ± SD PAR_EE_2_ | Mean ± SD PAR_3_ | Mean ± SD PAR_EE_3_ |
| 0.089  ± 0.026 | 0.101  ± 0.029 | 0.123  ± 0.042 | 0.137  ± 0.047 | 0.054  ± 0.032 | 0.067  ± 0.040 |
| PAR_1_ = pulp/tooth area ratio obtained from labiolingual radiograph; PAR_EE_1_ = pulp/tooth area ratio (excluding enamel) obtained from labiolingual radiograph; PAR_2_ = pulp/tooth area ratio obtained from mesiodistal radiograph; PAR_EE_2_ = pulp/tooth area ratio (excluding enamel) obtained from mesiodistal radiograph; PAR_3_ = pulp/tooth area ratio obtained from stereomicroscopic tooth section image; PAR_EE_3_ = pulp/tooth area ratio (excluding enamel) obtained from stereomicroscopic tooth section image; SD = standard deviation. | | | | | |

**Table S3** Best-subsets regression results for all candidate models, excluding those with high variance inflation factor values (VIF > 5). Models are grouped and ranked separately based on the number of predictor variables (k) they contain. Within each size group (k), they are ordered based on performance – from top (best) to bottom (worst) – as determined by selection criteria values

| k | Model | Performance/selection criteria | | | | |
| --- | --- | --- | --- | --- | --- | --- |
|  |  | *R*^2^-adjusted | SEE | AIC_c_ | BIC | *C_p_* |
| 1 | Age = f(PAR_EE_3_) | 0.75 | 9.88 | 390.25 | 395.60 | 5.49 |
|  | Age = f(PAR_3_) | 0.72 | 10.53 | 396.86 | 402.22 | 12.74 |
|  | Age = f(PAR_EE_1_) | 0.64 | 11.90 | 409.55 | 414.91 | 29.53 |
|  | Age = f(PAR_1_) | 0.64 | 12.03 | 410.74 | 416.10 | 31.32 |
|  | Age = f(PAR_EE_2_) | 0.42 | 15.11 | 434.46 | 439.81 | 77.16 |
|  | Age = f(PAR_2_) | 0.41 | 15.33 | 435.91 | 441.27 | 80.72 |
| 2 | Age = f(PAR_EE_1_, PAR_EE_3_) | 0.77 | 9.65 | 389.11 | 396.06 | 4.01 |
|  | Age = f(PAR_1_, PAR_EE_3_) | 0.76 | 9.70 | 389.63 | 396.58 | 4.52 |
|  | Age = f(PAR_EE_2_, PAR_EE_3_) | 0.75 | 9.93 | 392.05 | 399.00 | 6.92 |
|  | Age = f(PAR_2_, PAR_EE_3_) | 0.75 | 9.94 | 392.21 | 399.16 | 7.08 |
|  | Age = f(PAR_EE_1_, PAR_3_) | 0.74 | 10.09 | 393.77 | 400.73 | 8.71 |
|  | Age = f(PAR_1_, PAR_3_) | 0.74 | 10.20 | 394.88 | 401.84 | 9.89 |
|  | Age = f(PAR_EE_2_, PAR_3_) | 0.72 | 10.53 | 398.18 | 405.13 | 13.55 |
|  | Age = f(PAR_2_, PAR_3_) | 0.72 | 10.56 | 398.51 | 405.46 | 13.92 |
|  | Age = f(PAR_EE_1_, PAR_EE_2_) | 0.65 | 11.76 | 409.65 | 416.61 | 28.25 |
|  | Age = f(PAR_EE_1_, PAR_2_) | 0.65 | 11.78 | 409.82 | 416.77 | 28.48 |
|  | Age = f(PAR_1_, PAR_EE_2_) | 0.64 | 11.88 | 410.68 | 417.64 | 29.73 |
|  | Age = f(PAR_1_, PAR_2_) | 0.64 | 11.92 | 411.03 | 417.98 | 30.24 |
| 3 | Age = f(PAR_EE_1_, PAR_EE_2_, PAR_EE_3_) | 0.76 | 9.75 | 391.50 | 399.95 | 5.96 |
|  | Age = f(PAR_EE_1_, PAR_2_, PAR_EE_3_) | 0.76 | 9.75 | 391.54 | 399.99 | 5.99 |
|  | Age = f(PAR_1_, PAR_EE_2_, PAR_EE_3_) | 0.76 | 9.79 | 392.00 | 400.45 | 6.44 |
|  | Age = f(PAR_1_, PAR_2_, PAR_EE_3_) | 0.76 | 9.80 | 392.05 | 400.50 | 6.49 |
|  | Age = f(PAR_EE_1_, PAR_EE_2_, PAR_3_) | 0.74 | 10.18 | 396.08 | 404.54 | 10.56 |
|  | Age = f(PAR_EE_1_, PAR_2_, PAR_3_) | 0.74 | 10.19 | 396.16 | 404.62 | 10.64 |
|  | Age = f(PAR_1_, PAR_EE_2_, PAR_3_) | 0.73 | 10.29 | 397.12 | 405.57 | 11.65 |
|  | Age = f(PAR_1_, PAR_2_, PAR_3_) | 0.73 | 10.30 | 397.24 | 405.69 | 11.78 |
| STSI = stereomicroscopic tooth section image; LR = labiolingual radiograph; MR = mesiodistal radiograph; PAR_1_ = pulp/tooth area ratio obtained from labiolingual radiograph; PAR_EE_1_ = pulp/tooth area ratio (excluding enamel) obtained from labiolingual radiograph; PAR_2_ = pulp/tooth area ratio obtained from mesiodistal radiograph; PAR_EE_2_ = pulp/tooth area ratio (excluding enamel) obtained from mesiodistal radiograph; PAR_3_ = pulp/tooth area ratio obtained from stereomicroscopic tooth section image; PAR_EE_3_ = pulp/tooth area ratio (excluding enamel) obtained from stereomicroscopic tooth section image; *R*^2^-adjusted = adjusted coefficient of determination; SEE = standard error of the estimate; AIC_c_ = Akaike information criterion adjusted based on sample size; BIC = Bayesian information criterion; *C_p_* = Mallows’ prediction criterion. | | | | | | |

**Table S4** Analysis of variance (ANOVA) table outputs for each selected age estimation model

| Model | Source | df | SS | MS | *F*-statistic | *p* |
| --- | --- | --- | --- | --- | --- | --- |
| f(PAR_1_) | Regression | 1 | 12991.27 | 12991.27 | 89.73 | < 0.001 |
|  | Residual | 50 | 7239.04 | 144.78 |  |  |
|  | Total | 51 | 20230.31 |  |  |  |
| f(PAR_EE_1_) | Regression | 1 | 13155.04 | 13155.04 | 92.97 | < 0.001 |
|  | Residual | 50 | 7075.26 | 141.51 |  |  |
|  | Total | 51 | 20230.31 |  |  |  |
| f(PAR_2_) | Regression | 1 | 8483.57 | 8483.57 | 36.11 | < 0.001 |
|  | Residual | 50 | 11746.74 | 234.93 |  |  |
|  | Total | 51 | 20230.31 |  |  |  |
| f(PAR_EE_2_) | Regression | 1 | 8808.33 | 8808.33 | 38.56 | < 0.001 |
|  | Residual | 50 | 11421.98 | 228.44 |  |  |
|  | Total | 51 | 20230.31 |  |  |  |
| f(PAR_3_) | Regression | 1 | 14687.00 | 14687.00 | 132.47 | < 0.001 |
|  | Residual | 50 | 5543.31 | 110.87 |  |  |
|  | Total | 51 | 20230.31 |  |  |  |
| f(PAR_EE_3_) | Regression | 1 | 15349.01 | 15349.01 | 157.22 | < 0.001 |
|  | Residual | 50 | 4881.30 | 97.63 |  |  |
|  | Total | 51 | 20230.31 |  |  |  |
| f(PAR_EE_1_, PAR_EE_3_) | Regression | 2 | 15666.05 | 7833.03 | 84.09 | < 0.001 |
|  | Residual | 49 | 4564.26 | 93.15 |  |  |
|  | Total | 51 | 20230.31 |  |  |  |
| f(PAR_1_, PAR_EE_3_) | Regression | 2 | 15620.24 | 7810.12 | 83.01 | < 0.001 |
|  | Residual | 49 | 4610.06 | 94.08 |  |  |
|  | Total | 51 | 20230.31 |  |  |  |
| f(PAR_EE_1_, PAR_3_) | Regression | 2 | 15237.65 | 7618.83 | 74.77 | < 0.001 |
|  | Residual | 49 | 4992.65 | 101.89 |  |  |
|  | Total | 51 | 20230.31 |  |  |  |
| f(PAR_1_, PAR_3_) | Regression | 2 | 15130.14 | 7565.07 | 72.68 | < 0.001 |
|  | Residual | 49 | 5100.16 | 104.09 |  |  |
|  | Total | 51 | 20230.31 |  |  |  |
| f(PAR_EE_1_, PAR_EE_2_) | Regression | 2 | 13454.38 | 6727.19 | 48.65 | < 0.001 |
|  | Residual | 49 | 6775.93 | 138.28 |  |  |
|  | Total | 51 | 20230.31 |  |  |  |
| f(PAR_EE_1_, PAR_2_) | Regression | 2 | 13433.16 | 6716.58 | 48.42 | < 0.001 |
|  | Residual | 49 | 6797.15 | 138.72 |  |  |
|  | Total | 51 | 20230.31 |  |  |  |
| f(PAR_1_, PAR_EE_2_) | Regression | 2 | 13318.90 | 6659.45 | 47.21 | < 0.001 |
|  | Residual | 49 | 6911.41 | 141.05 |  |  |
|  | Total | 51 | 20230.31 |  |  |  |
| f(PAR_1_, PAR_2_) | Regression | 2 | 13272.88 | 6636.44 | 46.74 | < 0.001 |
|  | Residual | 49 | 6957.42 | 141.99 |  |  |
|  | Total | 51 | 20230.31 |  |  |  |
| PAR_1_ = pulp/tooth area ratio obtained from labiolingual radiograph; PAR_EE_1_ = pulp/tooth area ratio (excluding enamel) obtained from labiolingual radiograph; PAR_2_ = pulp/tooth area ratio obtained from mesiodistal radiograph; PAR_EE_2_ = pulp/tooth area ratio (excluding enamel) obtained from mesiodistal radiograph; PAR_3_ = pulp/tooth area ratio obtained from stereomicroscopic tooth section image; PAR_EE_3_ = pulp/tooth area ratio (excluding enamel) obtained from stereomicroscopic tooth section image; df = degrees of freedom; SS = sum of squares; MS = mean square. | | | | | | |

**Table S5**  Coefficients table outputs for each selected age estimation model

| Model | Predictor | Unstandardised coefficient | | Standardised coefficient | *t*-statistic | *p* | 95% CI for *B* |
| --- | --- | --- | --- | --- | --- | --- | --- |
|  |  | *B* | SE |  |  |  |  |
| f(PAR_1_) | Constant | 118.29 | 6.13 |  | 19.29 | < 0.001 | (105.97, 130.60) |
|  | PAR_1_ | -625.44 | 66.03 | -0.80 | -9.47 | < 0.001 | (-758.06, -492.82) |
| f(PAR_EE_1_) | Constant | 117.77 | 5.98 |  | 19.71 | < 0.001 | (105.77, 129.77) |
|  | PAR_EE_1_ | -548.95 | 56.93 | -0.81 | -9.64 | < 0.001 | (-663.31, -434.60) |
| f(PAR_2_) | Constant | 100.24 | 6.65 |  | 15.08 | < 0.001 | (86.88, 113.59) |
|  | PAR_2_ | -307.84 | 51.23 | -0.65 | -6.01 | < 0.001 | (-410.74, -204.95) |
| f(PAR_EE_2_) | Constant | 100.82 | 6.54 |  | 15.43 | < 0.001 | (87.70, 113.95) |
|  | PAR_EE_2_ | -281.03 | 45.26 | -0.66 | -6.21 | < 0.001 | (-371.93, -190.12) |
| f(PAR_3_) | Constant | 91.47 | 2.92 |  | 31.34 | < 0.001 | (85.60, 97.33) |
|  | PAR_3_ | -534.64 | 46.45 | -0.85 | -11.51 | < 0.001 | (-627.94, -441.34) |
| f(PAR_EE_3_) | Constant | 91.83 | 2.72 |  | 33.78 | < 0.001 | (86.37, 97.29) |
|  | PAR_EE_3_ | -439.05 | 35.02 | -0.87 | -12.54 | < 0.001 | (-509.38, -368.72) |
| f(PAR_EE_1_, PAR_EE_3_) | Constant | 101.32 | 5.79 |  | 17.49 | < 0.001 | (89.68, 112.96) |
|  | PAR_EE_1_ | -161.82 | 87.71 | -0.24 | -1.84 | 0.071 | (-338.08, 14.44) |
|  | PAR_EE_3_ | -337.20 | 64.95 | -0.67 | -5.19 | < 0.001 | (-467.71, -206.69) |
| f(PAR_1_, PAR_EE_3_) | Constant | 100.84 | 5.94 |  | 16.97 | < 0.001 | (88.90, 112.79) |
|  | PAR_1_ | -171.53 | 101.03 | -0.22 | -1.70 | 0.096 | (-374.55, 31.48) |
|  | PAR_EE_3_ | -344.89 | 65.25 | -0.68 | -5.29 | < 0.001 | (-476.01, -213.78) |
| f(PAR_EE_1_, PAR_3_) | Constant | 103.68 | 5.95 |  | 17.42 | < 0.001 | (91.72, 115.64) |
|  | PAR_EE_1_ | -208.15 | 89.54 | -0.31 | -2.32 | 0.024 | (-388.08, -28.22) |
|  | PAR_3_ | -373.11 | 82.53 | -0.59 | -4.52 | < 0.001 | (-538.96, -207.27) |
| f(PAR_1_, PAR_3_) | Constant | 102.88 | 6.21 |  | 16.56 | < 0.001 | (90.40, 115.36) |
|  | PAR_1_ | -218.35 | 105.82 | -0.28 | -2.06 | 0.044 | (-431.01, -5.70) |
|  | PAR_3_ | -385.67 | 85.08 | -0.62 | -4.53 | < 0.001 | (-556.64, -214.70) |
| f(PAR_EE_1_, PAR_EE_2_) | Constant | 119.39 | 6.01 |  | 19.87 | < 0.001 | (107.31, 131.46) |
|  | PAR_EE_1_ | -464.89 | 80.20 | -0.68 | -5.80 | < 0.001 | (-626.06, -303.71) |
|  | PAR_EE_2_ | -73.82 | 50.18 | -0.17 | -1.47 | 0.148 | (-174.66, 27.01) |
| f(PAR_EE_1_, PAR_2_) | Constant | 119.48 | 6.04 |  | 19.78 | < 0.001 | (107.34, 131.62) |
|  | PAR_EE_1_ | -470.92 | 78.84 | -0.69 | -5.97 | < 0.001 | (-629.34, -312.49) |
|  | PAR_2_ | -77.95 | 55.05 | -0.16 | -1.42 | 0.163 | (-188.58, 32.68) |
| f(PAR_1_, PAR_EE_2_) | Constant | 119.84 | 6.14 |  | 19.52 | < 0.001 | (107.51, 132.18) |
|  | PAR_1_ | -524.76 | 92.80 | -0.67 | -5.66 | < 0.001 | (-711.24, -338.28) |
|  | PAR_EE_2_ | -77.17 | 50.64 | -0.18 | -1.52 | 0.134 | (-178.93, 24.58) |
| f(PAR_1_, PAR_2_) | Constant | 119.84 | 6.17 |  | 19.41 | < 0.001 | (107.44, 132.25) |
|  | PAR_1_ | -534.30 | 92.00 | -0.68 | -5.81 | < 0.001 | (-719.18, -349.43) |
|  | PAR_2_ | -78.91 | 56.03 | -0.17 | -1.41 | 0.165 | (-191.52, 33.69) |
| PAR_1_ = pulp/tooth area ratio obtained from labiolingual radiograph; PAR_EE_1_ = pulp/tooth area ratio (excluding enamel) obtained from labiolingual radiograph; PAR_2_ = pulp/tooth area ratio obtained from mesiodistal radiograph; PAR_EE_2_ = pulp/tooth area ratio (excluding enamel) obtained from mesiodistal radiograph; PAR_3_ = pulp/tooth area ratio obtained from stereomicroscopic tooth section image; PAR_EE_3_ = pulp/tooth area ratio (excluding enamel) obtained from stereomicroscopic tooth section image; *B* = unstandardised coefficient value; SE = standard error; CI = confidence interval. | | | | | | | |

**Table S6** Assessing homoscedasticity and normality of the standardised residuals for each selected age estimation model using Breusch-Pagan and Shapiro-Wilk tests

| Model | Breusch-Pagan test | | | Shapiro-Wilk test | | |
| --- | --- | --- | --- | --- | --- | --- |
|  | X^2^ | df | *p* | *W*-statistic | df | *p* |
| f(PAR_1_) | 0.73 | 1 | 0.392 | 0.98 | 52 | 0.529 |
| f(PAR_EE_1_) | 0.78 | 1 | 0.377 | 0.98 | 52 | 0.640 |
| f(PAR_2_) | 2.60 | 1 | 0.107 | 0.99 | 52 | 0.940 |
| f(PAR_EE_2_) | 3.07 | 1 | 0.080 | 0.99 | 52 | 0.942 |
| f(PAR_3_) | 0.47 | 1 | 0.494 | 0.96 | 52 | 0.083 |
| f(PAR_EE_3_) | 0.31 | 1 | 0.576 | 0.96 | 52 | 0.102 |
| f(PAR_EE_1_, PAR_EE_3_) | 0.36 | 2 | 0.835 | 0.96 | 52 | 0.098 |
| f(PAR_1_, PAR_EE_3_) | 0.42 | 2 | 0.811 | 0.96 | 52 | 0.099 |
| f(PAR_EE_1_, PAR_3_) | 0.52 | 2 | 0.771 | 0.97 | 52 | 0.165 |
| f(PAR_1_, PAR_3_) | 0.47 | 2 | 0.791 | 0.97 | 52 | 0.150 |
| f(PAR_EE_1_, PAR_EE_2_) | 0.62 | 2 | 0.733 | 0.99 | 52 | 0.781 |
| f(PAR_EE_1_, PAR_2_) | 0.57 | 2 | 0.752 | 0.99 | 52 | 0.807 |
| f(PAR_1_, PAR_EE_2_) | 0.61 | 2 | 0.737 | 0.99 | 52 | 0.792 |
| f(PAR_1_, PAR_2_) | 0.60 | 2 | 0.741 | 0.99 | 52 | 0.780 |
| X^2^ = chi-square test statistic; df = degrees of freedom; PAR_1_ = pulp/tooth area ratio obtained from labiolingual radiograph; PAR_EE_1_ = pulp/tooth area ratio (excluding enamel) obtained from labiolingual radiograph; PAR_2_ = pulp/tooth area ratio obtained from mesiodistal radiograph; PAR_EE_2_ = pulp/tooth area ratio (excluding enamel) obtained from mesiodistal radiograph; PAR_3_ = pulp/tooth area ratio obtained from stereomicroscopic tooth section image; PAR_EE_3_ = pulp/tooth area ratio (excluding enamel) obtained from stereomicroscopic tooth section image. | | | | | | |

**Table S7** Leave-one-out cross-validation (LOOCV) parameters for assessing model accuracy and optimism bias. Models are listed in the same order as they appear in Table 4

| k | Image type | Model | *R*^2^_CV_ | Absolute residual parameters (years) | | | Prediction bias parameters | | |
| --- | --- | --- | --- | --- | --- | --- | --- | --- | --- |
|  |  |  |  | MAE_CV_ | SD | SEE_CV_ | ΔMAE (years) | ΔSEE (years) | Δ*R*^2^ |
| 1 | **STSI** | Age = f(PAR_EE_3_) | 0.74 | 7.45 | 6.77 | 10.17 | 0.24 | 0.29 | -0.02 |
|  |  | Age = f(PAR_3_) | 0.71 | 7.72 | 7.45 | 10.84 | 0.25 | 0.31 | -0.02 |
|  | **LR** | Age = f(PAR_EE_1_) | 0.63 | 9.89 | 7.07 | 12.32 | 0.37 | 0.42 | -0.02 |
|  |  | Age = f(PAR_1_) | 0.62 | 10.13 | 6.94 | 12.45 | 0.37 | 0.42 | -0.02 |
|  | **MR** | Age = f(PAR_EE_2_) | 0.40 | 12.31 | 9.32 | 15.64 | 0.44 | 0.52 | -0.04 |
|  |  | Age = f(PAR_2_) | 0.38 | 12.37 | 9.61 | 15.85 | 0.44 | 0.53 | -0.04 |
| 2 | **STSI**  **+**  **LR** | Age = f(PAR_EE_1_, PAR_EE_3_) | 0.75 | 7.35 | 6.76 | 10.15 | 0.38 | 0.50 | -0.02 |
|  |  | Age = f(PAR_1_, PAR_EE_3_) | 0.75 | 7.40 | 6.74 | 10.17 | 0.37 | 0.47 | -0.02 |
|  |  | Age = f(PAR_EE_1_, PAR_3_) | 0.73 | 7.83* | 6.96 | 10.65 | 0.43 | 0.56 | -0.02 |
|  |  | Age = f(PAR_1_, PAR_3_) | 0.72 | 7.88* | 7.01 | 10.72 | 0.41 | 0.52 | -0.03 |
|  | **LR**  **+**  **MR** | Age = f(PAR_EE_1_, PAR_EE_2_) | 0.63 | 9.67 | 7.40 | 12.41* | 0.54 | 0.65 | -0.04 |
|  |  | Age = f(PAR_EE_1_, PAR_2_) | 0.63 | 9.69 | 7.42 | 12.44* | 0.56 | 0.66 | -0.03 |
|  |  | Age = f(PAR_1_, PAR_EE_2_) | 0.62 | 9.70 | 7.55 | 12.52* | 0.53 | 0.65 | -0.04 |
|  |  | Age = f(PAR_1_, PAR_2_) | 0.62 | 9.74 | 7.57 | 12.57* | 0.54 | 0.66 | -0.04 |
| k = number of predictor variables in the model; STSI = stereomicroscopic tooth section image; LR = labiolingual radiograph; MR = mesiodistal radiograph; *R*^2^_CV_ = cross-validation coefficient of determination; MAE_CV_ = cross-validation mean absolute error; SD = standard deviation; SEE_CV_ = cross-validation standard error of the estimate; Δ*R*^2^ = *R*^2^_CV_ - *R*^2^ (in-sample coefficient of determination); ΔMAE = MAE_CV_ – MAE (in-sample mean absolute error); ∆SEE = SEE_CV_ – SEE (in-sample standard error of the estimate); PAR_1_ = pulp/tooth area ratio obtained from labiolingual radiograph; PAR_EE_1_ = pulp/tooth area ratio (excluding enamel) obtained from labiolingual radiograph; PAR_2_ = pulp/tooth area ratio obtained from mesiodistal radiograph; PAR_EE_2_ = pulp/tooth area ratio (excluding enamel) obtained from mesiodistal radiograph; PAR_3_ = pulp/tooth area ratio obtained from stereomicroscopic tooth section image; PAR_EE_3_ = pulp/tooth area ratio (excluding enamel) obtained from stereomicroscopic tooth section image. For multiple regression models, error values with * are higher compared to simpler subset models. | | | | | | | | | |
